# Underrepresentation of activating KIR gene expression in single-cell RNA-seq data is due to KIR gene misassignment

**DOI:** 10.1002/eji.202350590

**Published:** 2023-11-20

**Authors:** Eric Alves, Abha Chopra, Ramesh Ram, Jennifer Currenti, Spyros A. Kalams, Simon A. Mallal, Elizabeth J. Phillips, Silvana Gaudieri

**Affiliations:** 1 School of Human Sciences, University of Western Australia, Crawley, Western Australia, Australia; 2 Harry Perkins Institute of Medical Research, QEII Medical Centre, Nedlands, Western Australia, Australia; 3 Institute for Immunology and Infectious Diseases, Murdoch University, Murdoch, Western Australia, Australia; 4 School of Medicine, Curtin University, Bentley, Western Australia, Australia; 5 Department of Medicine, Vanderbilt University Medical Center, Nashville, Tennessee, United States of America

Killer immunoglobulin-like receptors (KIRs) are polymorphic genes expressed on NK cells and T cells [1, 2]. The KIR gene complex is polygenic and segregates into two haplotype groups, which exhibit variable associations with reproductive diseases (notably preeclampsia [3]), infectious diseases, autoimmune conditions and cancers [4]. The A-haplotype has a fixed gene content, consisting largely of inhibitory KIRs (KIR2D**L**/3D**L**), whereas the B-haplotype is more diverse and contains one or more genes encoding activating KIRs (KIR2D**S**/3D**S**). Advancements in single-cell RNA-sequencing (scRNA-seq) have enabled profiling of individual KIRs and identification of clonally expanded KIR^+^ cells in disease [5]. However, an analysis of 223 short-read scRNA-seq datasets, encompassing different human tissues and diseases (https://singlecell.broadinstitute.org/single_cell), found 42% (6/14) of functional KIRs (*KIR2DL5A/B*, *KIR2DS1/S2/S3/S5, KIR3DS1*) undetected.

The absence of these KIRs, characterizing the B-haplotype, suggests an inherent bias toward the A-haplotype in scRNA-seq. The omission of B-haplotype-associated KIRs likely stems from limitations in scRNA-seq analysis pipelines. We hypothesized that this discrepancy may be attributable to the GRCh37/38 references utilized for alignment. Indeed, only seven KIRs (*KIR2DL1/L3/L4*, *KIR2DS4*, *3DL1/L2/L3*) are present in the GRCh37/38 references, resulting in an inability to report the aforementioned six KIRs.

We postulated that transcripts from these six KIRs may be erroneously aligned to homologous KIR loci. Therefore, we performed allelic-resolution KIR genotyping on a single donor (Fig. 1A; Supporting Information 1 Table S1) and utilized matched peripheral blood mononuclear cells (PBMCs) for a representative scRNA-seq experiment using the 10× Genomics 5′ approach (Fig. 1B; Supporting Information 1 Materials and Methods). KIR-assigned reads were extracted following default sequence alignment using the GRCh38 reference with Ensembl v93 gene annotations in Cell Ranger.

It was observed that counts were not quantified for *KIR2DS4*, despite its inclusion in the GRCh38 reference. We elucidated that this default pipeline applies a filtered gene annotation that excludes pseudogenes (Fig. 1B) [6]. As *KIR2DS4* is classified as a ‘polymorphic pseudogene’ in Ensembl v93, this clarifies why *KIR2DS4* is only detectable when unfiltered or custom gene annotations are used [6]. Thus, the default pipeline exclusively quantified counts for six KIRs (*KIR2DL1/L3/L4*, *KIR3DL1/L2/L3*).

For this donor, our analysis confirmed the accurate assignment of reads mapped to *KIR2DL3*, *KIR2DL4* and *KIR3DL2*. Furthermore, the absence of counts for *KIR3DL3* aligns with the known rarity of *KIR3DL3*^+^ cells in PBMCs [7], suggesting that these are correctly assigned. However, polymorphisms were identified within transcripts mapped to *KIR3DL1* and *KIR2DL1* (Fig. 1C and D). Reads mapped to *KIR3DL1* exhibited polymorphisms associated with *KIR3DS1* (Fig. 1C), indicating that these transcripts correspond to *KIR3DS1* expression. Similarly, transcripts mapped to *KIR2DL1* displayed a combination of polymorphisms associated with *KIR2DL1*, *KIR2DS1* and *KIR2DS5* (Fig. 1D). These findings align with the donor’s KIR repertoire (Fig. 1A; Supporting Information 1 Table S1). This exemplifies that standard scRNA-seq workflows may overlook *KIR2DS4* and B-haplotype-associated KIR expression, which include activating KIRs involved in T cell activation [8], NK cell education [9] and bacterial [10, 11] or viral [12] recognition. Additionally, this study demonstrates that activating KIR transcripts may be misclassified as inhibitory KIRs, specifically *KIR3DL1* and *KIR2DL1*, leading to an overestimation of inhibitory KIR counts.

To rectify the loss and/or misclassification of activating KIR counts, reads were re-mapped to an amended GRCh38 reference incorporating all KIRs (14 functional genes and 2 pseudogenes), including separate *KIR3DL1* and *KIR3DS1* genes (Fig. 1E; Supporting Information 1 Materials and Methods). Doing so accurately reassigned transcripts in accordance with KIR genotyping (Fig. 1F and G). Notably, transcripts initially attributed to *KIR3DL1* were appropriately reclassified as *KIR3DS1*. Similarly, previously assigned *KIR2DL1* transcripts were redistributed among *KIR2DL1/S1/S5*. Moreover, *KIR2DL5* counts were now quantified, which were not initially misassigned to any KIRs during default alignment, but rather, originated from unmapped reads.

A minor fraction of transcripts (2.6%) aligned to *KIR2DL2*, a result inconsistent with the donor’s KIR genotype. Incorporation of the donor’s KIR allele sequences into the reference did not resolve this misassignment. Furthermore, comparing total counts across alignments, we observed a 2.9% reduction in KIR-specific counts upon realignment to the KIR-modified reference. This reduction stemmed from an increased number of discarded reads owing to multi-mapping (i.e. reads mapping to multiple loci). The high homology within exons (1/2/6/7/8) between KIRs highlights regions where misassignment and/or multi-mapping might occur (Supporting Information 1 Figs. S1–8; Supporting Information 2). Nevertheless, realignment to the KIR-modified reference enhanced profiling of KIRs, elucidating complex co-expression patterns, such as *KIR3DS1*^+^*KIR3DL2*^+^*KIR2DL3*^+^*KIR2DS1*^+^*KIR2DL4*^+^*KIR2DL1*^+^ cells (Fig. 1H), which likely go unnoticed with standard scRNA-seq workflows.

To validate the issue of KIR misassignment and improvements achieved by mapping to the KIR-modified GRCh38 reference, scRNA-seq was conducted on PBMCs from an additional six donors with diverse KIR repertoires (Supporting Information 1 Table S1, Supporting Information 1 Fig. S9). This confirmed the correct assignment of reads for *KIR2DL4* and *KIR3DL2* with the default Cell Ranger pipeline (Supporting Information 1 Fig. S9). Similarly, the absence of *KIR3DL3* expression in PBMCs among all donors is as expected [7] and suggests that the misassignment of non-*KIR3DL3* transcripts to the *KIR3DL3* locus is unlikely. As for donor 1, counts absent in the realigned data were associated with transcripts discarded due to multi-mapping, and therefore, the incorporation of all KIR genes in the GRCh38 reference may reduce total transcript quantification. However, although reads were accurately assigned to *KIR2DL3* for donor 1 using the default workflow, reads were incorrectly assigned to *KIR2DL3* for donors 2 and 7. Upon re-mapping to the KIR-modified reference, these reads were correctly reassigned to the genotype-consistent *KIR2DL2/S2*, which underscores the improved detection of KIR diversity using a KIR-modified reference.

Notably, *KIR2DS4* expression was only observed in three donors (3, 6 and 7). Given that all six donors’ *KIR2DS4* allele sequences (*001, *003, *004, *010 and *016) are identical at most exons (1/2/6/7/8), near-identical at remaining exons (3/4/5) and sufficiently unique (Supporting Information 1 Figs. S1–8) from other KIRs to minimize mismapping, it suggests that absence of *KIR2DS4* in samples from donors 2, 4 and 5 is accurate.

During realignment, a minor portion of reads were misassigned. Namely, 2.2% and 4% of transcripts from donors 3 and 7, respectively, were erroneously assigned to *KIR3DS1*, instead of *KIR3DL1* (Supporting Information 1 Fig. S9). This stems from the high homology between the donors’ *KIR3DL1* allele sequences (*007, *008, *015 and *020) and the *KIR3DS1* reference. Specifically, the KIR3DL1*007/008/015/020 alleles are identical to the *KIR3DS1* reference at exons 1/2, leading to transcript misalignment. This also clarifies why donor 4 (KIR3DL1*007) only exhibits *KIR3DS1* transcripts. However, the absence of *KIR3DL1* expression in donors 5 and 6, both carrying the KIR3DL1*005 allele (similar to donor 2), which more closely resembles the *KIR3DL1* reference, implies that the lack of *KIR3DL1* abundance is true in these samples. Ultimately, misassignment of *KIR3DL1/S1* transcripts using both pipelines highlights the challenge of assigning *KIR3DL1/S1* expression in the absence of allelic-resolution KIR genotyping. Consequently, it is advisable to interpret *KIR3DL1* expression in existing scRNA-seq data as combined *KIR3DL1/S1* expression, unless genotyping can confirm the presence of one form and/or the accuracy of *KIR3DL1* allele sequence alignment can be verified.

In cases where genotyping is unavailable and comprehensive KIR alignment tools [13] are unfeasible, aligning scRNA-seq data to a KIR-modified GRCh38 reference may enhance KIR expression profiling. However, despite the improvements achieved with this approach, the high homology among KIRs (particularly in exons 1/2/6/7/8) still poses challenges in accurately detecting KIR expression. For precise examination of KIR repertoire, long-read sequencing technology is recommended. This technology covers multiple KIR exons and allele-specific polymorphisms, enabling accurate distinction between KIR alleles when combined with allelic-resolution KIR genotyping [14].

Although efforts to capture global genetic heterogeneity in genomic studies are advancing [15], these findings emphasize the ongoing challenge in accurately representing human diversity. By presenting a scenario where standard scRNA-seq workflows inaccurately assigned transcripts within the highly homologous, yet functionally distinct, KIR gene family, this study underscores how such inaccuracies can obscure critical biological insights or lead to erroneous inferences. In conclusion, our findings emphasize the need for caution when interpreting KIR expression in scRNA-seq without confirmatory methods and highlight the importance of carefully considering analysis pipelines and potential biases in reference genomes.

## Supplementary Material

Supplementary Information 2

Supplementary Information 1

## Figures and Tables

**Figure 1.**
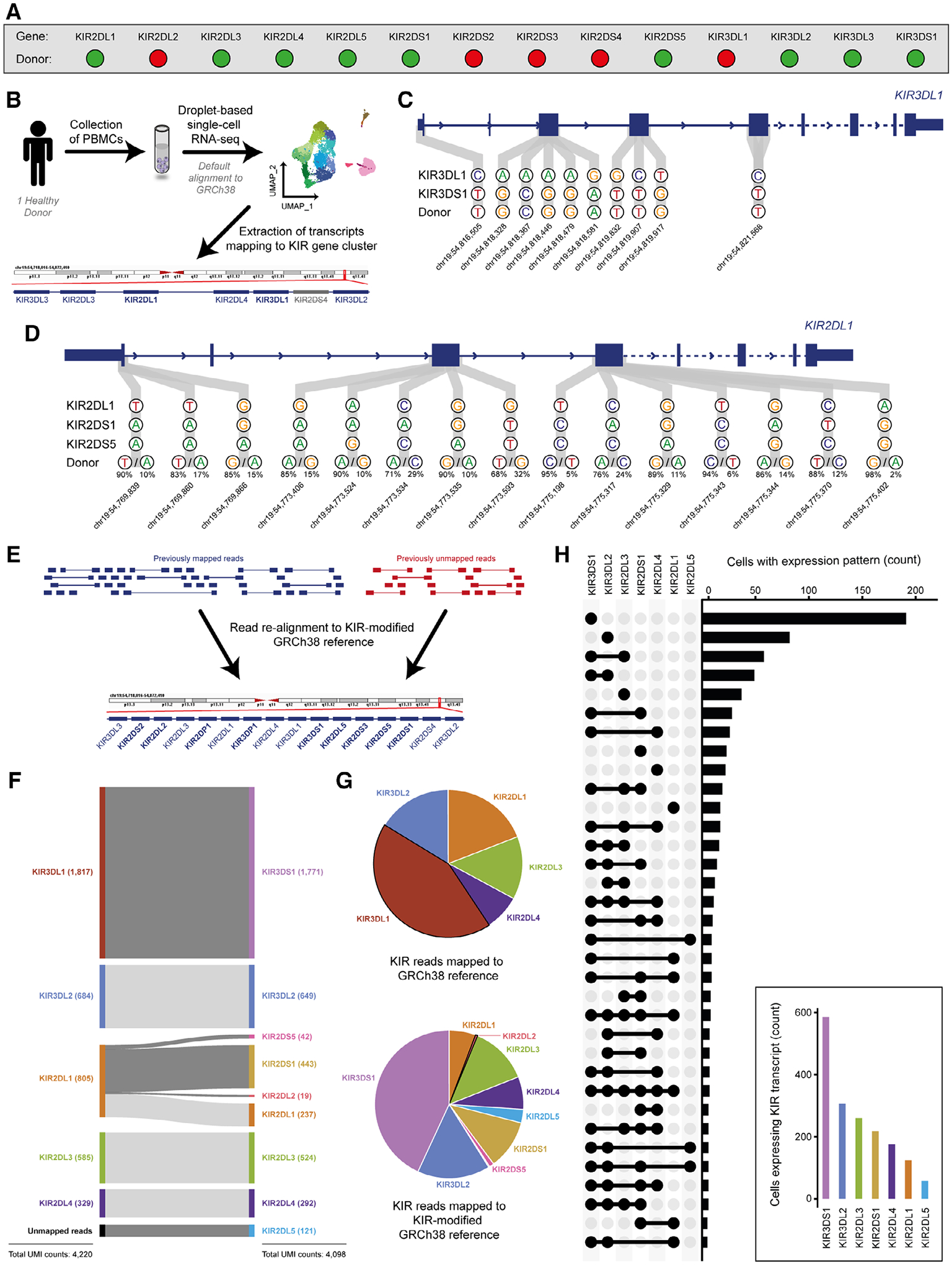
Activating killer immunoglobulin-like receptor (KIR) transcripts map to inhibitory KIR loci with default single-cell RNA-sequencing (scRNA-seq) alignment. (A) KIR genotyping of donor 1. Green, gene is present; red, gene is absent. (B) Schematic for evaluating the accuracy of KIR-assigned reads following 10× Genomics 5′ scRNA-seq with default Cell Ranger alignment. (C) *KIR3DL1* counts reflect mismapped *KIR3DS1* reads. (D) *KIR2DL1* counts reflect *KIR2DL1*, *KIR2DS1* and *KIR2DS5* reads. (E) Schematic for realigning scRNA-seq reads to the KIR-modified GRCh38 reference. (F) Re-distribution of KIR counts following realignment. (G) Proportion of reads mapping to KIR loci based on the reference used for alignment. Sections with black outline reflect incorrectly mapped reads to genes not in the donor’s genotype. (H) Most prevalent KIR co-expression patterns following realignment.

## Data Availability

The scRNA-seq data generated from donor 1 are available here: https://www.ncbi.nlm.nih.gov/, PRJNA945569. The scRNA-seq data generated from donors 2–7 are available from the corresponding author upon request. The KIR-modified GRCh38 reference and KIR gene sequences utilized in the reference modification are available here: https://doi.org/10.5281/zenodo.7762015.
