# Supplementary material for: Underrepresentation of activating KIR gene expression in single-cell RNA-seq data is due to KIR gene misassignment
Source: Eur J Immunol. Author manuscript; Available in PMC 2025 May 18. (PMC12085302; doi:10.1002/eji.202350590)
Supplement: Supplementary Information 1 [file NIHMS2080589-supplement-Supplementary_Information_1.docx]

# Underrepresentation of activating KIR gene expression in single-cell RNA-seq data is due to KIR gene misassignment

Eric Alves^1,2^, Abha Chopra^3^, Ramesh Ram^3^, Jennifer Currenti^2,4^, Spyros A. Kalams^5^, Simon A. Mallal^3,5^, Elizabeth J. Phillips^3,5^, Silvana Gaudieri^1,3,5*^

^1^School of Human Sciences, University of Western Australia, Crawley, Western Australia, Australia

^2^Harry Perkins Institute of Medical Research, QEII Medical Centre, Nedlands, Western Australia, Australia

^3^Institute for Immunology and Infectious Diseases, Murdoch University, Murdoch, Western Australia, Australia

^4^School of Medicine, Curtin University, Bentley, Western Australia, Australia

^5^Department of Medicine, Vanderbilt University Medical Center, Nashville, Tennessee, United States of America

**^*^**Address correspondence to Associate Professor Silvana Gaudieri, email: silvana.gaudieri@uwa.edu.au

# Materials and Methods

*Subject material, ethics and consent*

Whole blood was obtained with written informed consent from seven donors from Vanderbilt University Medical Center. Peripheral blood mononuclear cells (PBMCs) were isolated from whole blood by Ficoll density gradient separation and cryopreserved. Genomic DNA was extracted from PBMCs using the QIAGEN Extraction Kit (QIAGEN, NRW, Germany), as per the manufacturer’s instructions.

*KIR genotyping*

KIR allelic sequencing on genomic DNA was performed as previously reported [1]. Briefly, uniquely indexed primers designed to target KIR exons 3, 4 and 5 (D0, D1 and D2 domains) were pooled in a multiplexed PCR assay. Amplicons spanning the full exon were combined in appropriate volumes for balanced read coverage and sequenced on the Illumina MiSeq platform using a 2x300 bp paired-end chemistry kit (Illumina, CA, USA). Paired-end reads were quality filtered, demultiplexed and merged based on Q30 scores. Single reads were aligned to a reference sequence containing all KIR genes using CLCbio genomics workbench (QIAGEN Bioinformatics). Allele calls were based on the current IPD-KIR Database [2].

*Single-cell RNA sequencing (scRNA-seq)*

scRNA-seq was conducted using the 10x Genomics Chromium approach, as previously described [3]. Briefly, ten thousand resting PBMCs were loaded onto the Chromium Single Cell 5’ assay using the Chromium Single Cell v2 5’ Chemistry Library (10x Genomics, CA, USA), as per the manufacturer’s protocol. The cDNA libraries were sequenced on an Illumina NovaSeq S4 using a 2x150 bp paired-end chemistry kit (Illumina, CA, USA).

Raw sequencing reads were aligned using default settings to the GRCh38 (v3.0.0) human genome reference with Ensembl v93 gene annotations in Cell Ranger (v7.1.0). The same raw sequencing reads were similarly aligned to a KIR-modified GRCh38 (v3.0.0) human genome reference generated by masking the existing KIR genes and inserting new KIR sequences representing all possible KIR genes. Unique molecular identifier (UMI) counts for the KIR genes and KIR-assigned reads from both outputs were extracted. KIR genes with ≥ 10 UMIs were analyzed in downstream analyses in R (v4.1.0; Seurat package v4.1.0 [4]) and Integrative Genomics Viewer [5].

*Phylogenetic analyses*

Examination of nucleotide distances between KIR alleles was performed in MEGA X [6]. KIR allele sequences were extracted from the IPD-KIR Database [2] and separated by exon. Phylogenetic trees were constructed for each exon using the Neighbor-Joining method [7], with evolutionary distances computed using the number of nucleotide base differences per unique exon sequence, which represent the number of nucleotide mismatches utilized in scRNA-seq read alignment. Trees are drawn to scale, with branch lengths in the same units as those of the evolutionary distances (nucleotide base differences). Label notation refers to the KIR gene, with the number of alleles matching the unique exon sequence in brackets. KIR genes with identical exon sequences are combined where applicable.

# References

1. Margolis DJ, Mitra N, Hoffstad OJ, Berna R, Kim BS, Chopra A, et al. Association of KIR2DL5, KIR2DS5, and KIR2DS1 allelic variation and atopic dermatitis. Scientific Reports. 2023;13(1):1730. doi: 10.1038/s41598-023-28847-y.

2. Barker DJ, Maccari G, Georgiou X, Cooper MA, Flicek P, Robinson J, et al. The IPD-IMGT/HLA Database. Nucleic Acids Research. 2022;51(D1):D1053-D60. doi: 10.1093/nar/gkac1011.

3. Currenti J, Law BMP, Qin K, John M, Pilkinton MA, Bansal A, et al. Cross-reactivity to mutated viral immune targets can influence CD8+ T cell functionality: An alternative viral adaptation strategy. Frontiers in Immunology. 2021;12. doi: 10.3389/fimmu.2021.746986.

4. Hao Y, Hao S, Andersen-Nissen E, Mauck WM, Zheng S, Butler A, et al. Integrated analysis of multimodal single-cell data. Cell. 2021;184(13):3573-87.e29. doi: 10.1016/j.cell.2021.04.048.

5. Thorvaldsdóttir H, Robinson JT, Mesirov JP. Integrative Genomics Viewer (IGV): high-performance genomics data visualization and exploration. Briefings in Bioinformatics. 2012;14(2):178-92. doi: 10.1093/bib/bbs017.

6. Kumar S, Stecher G, Li M, Knyaz C, Tamura K. MEGA X: Molecular Evolutionary Genetics Analysis across Computing Platforms. Molecular Biology and Evolution. 2018;35(6):1547-9. doi: 10.1093/molbev/msy096.

7. Saitou N, Nei M. The neighbor-joining method: a new method for reconstructing phylogenetic trees. Molecular Biology and Evolution. 1987;4(4):406-25. doi: 10.1093/oxfordjournals.molbev.a040454.

# Supplementary Table

**Table S1. Allelic-resolution KIR genotyping of donors.**

| ***Donor*** | ***KIR*** | | | | | | | | | | | | | | | |
| --- | --- | --- | --- | --- | --- | --- | --- | --- | --- | --- | --- | --- | --- | --- | --- | --- |
|  | ***2DL1*** | ***2DL2*** | ***2DL3*** | ***2DL4*** | ***2DL5*** | ***2DP1*** | ***2DS1*** | ***2DS2*** | ***2DS3*** | ***2DS4*** | ***2DS5*** | ***3DL1*** | ***3DL2*** | ***3DL3*** | ***3DP1*** | ***3DS1*** |
| 1 | 001, 003 |  | 001 | 005 | 001 | 002, 003 | 002, 012 |  |  |  | 002 |  | 006 | 001, 010 | 001, 005 | 010 |
| 2 | 012 | 003, 006 |  | 002, 005 | 001 | 001 |  | 001 | 001 | 003 | 005 | 001, 005 | 001 | 001, 040 | 001, 003 |  |
| 3 | 003 | 001 | 001 | 001 |  | 002 |  | 001 |  | 001 |  | 015, 020 | 009 | 001, 002 | 001 |  |
| 4 | 001, 012 | 001 | 001 | 005, 006 | 001, 002 | 001, 003 | 002 | 001 | 008 | 004 | 002 | 007 | 006, 008 | 001, 006 | 003, 005 | 010 |
| 5 | 001 | 001 | 001 | 005 |  | 001, 003 | 002 | 001 | 001 | 010 | 002 | 005 | 001, 006 | 001 | 001, 005 | 010 |
| 6 | 003 | 003 | 001 | 001, 005 |  | 002 |  | 001 |  | 001, 016 |  | 005, 061 | 001, 002 | 001 | 001 |  |
| 7 | 042 | 001, 003 |  | 001, 006 | 002 | 012 |  | 001 | 001 | 001 |  | 007, 008 | 008, 019 | 001, 002 | 001, 003 |  |

# Supplementary Figures


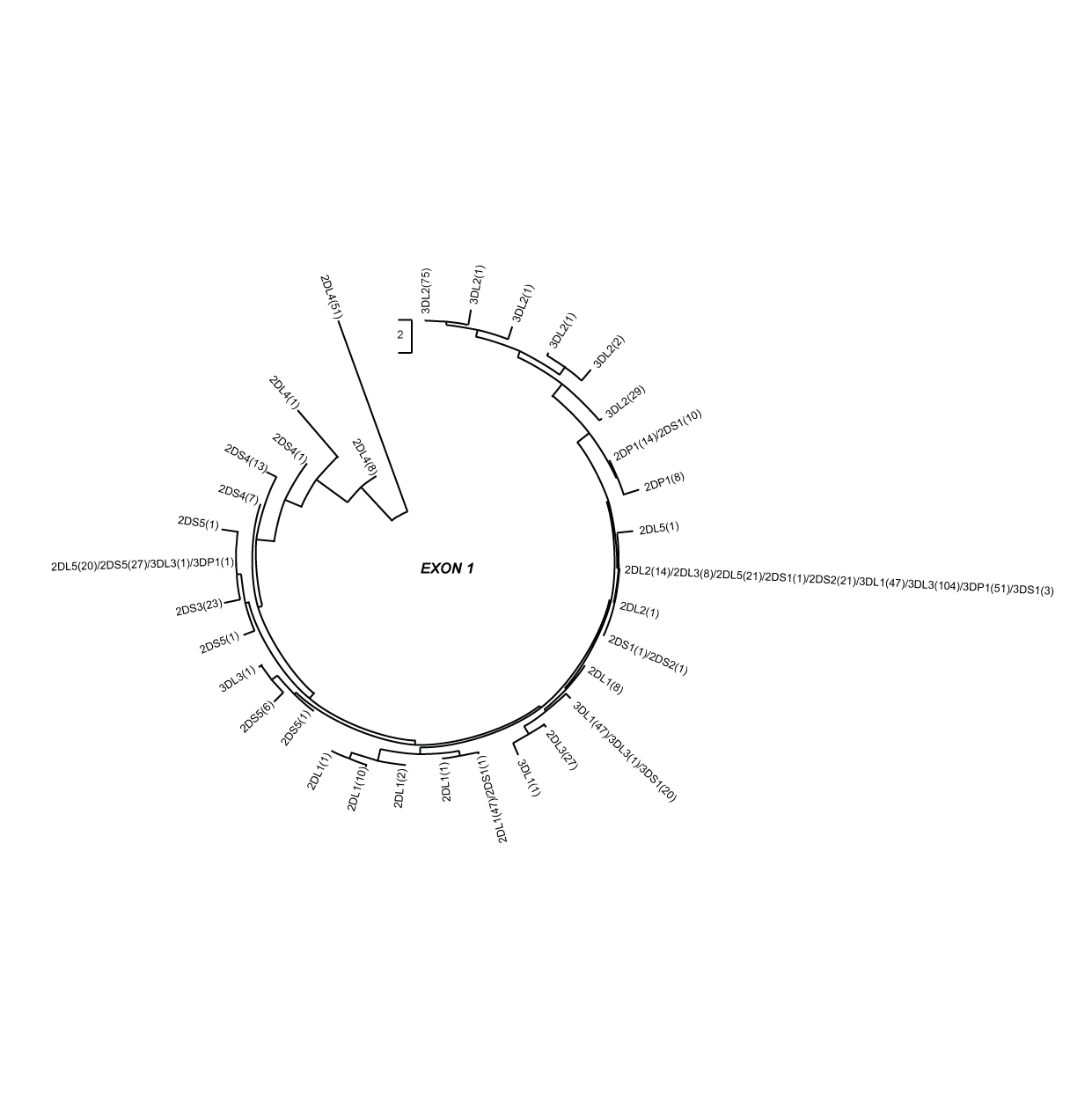


**Figure S1. Phylogenetic analysis of KIR allele sequence identity at exon 1.**


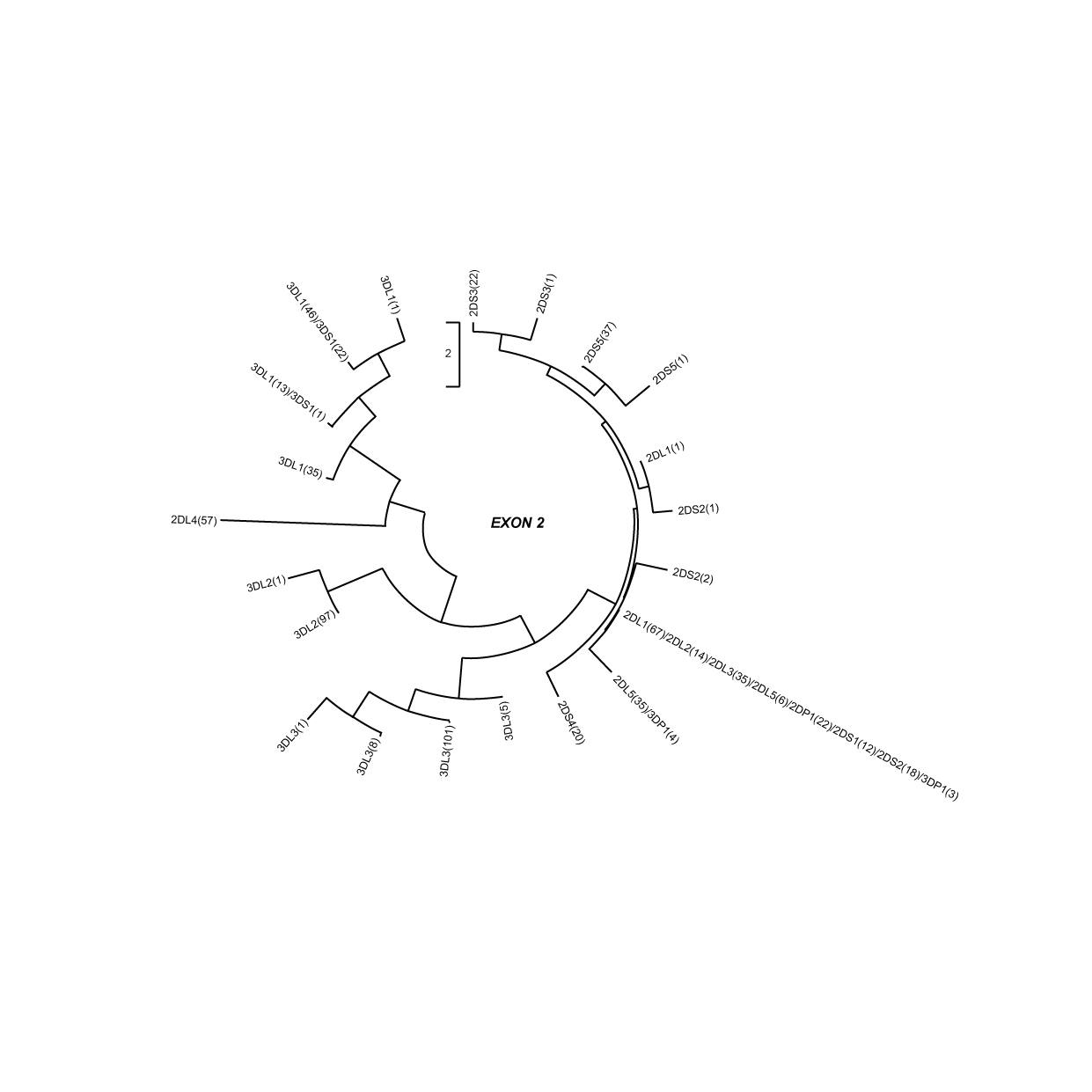


**Figure S2. Phylogenetic analysis of KIR allele sequence identity at exon 2.**


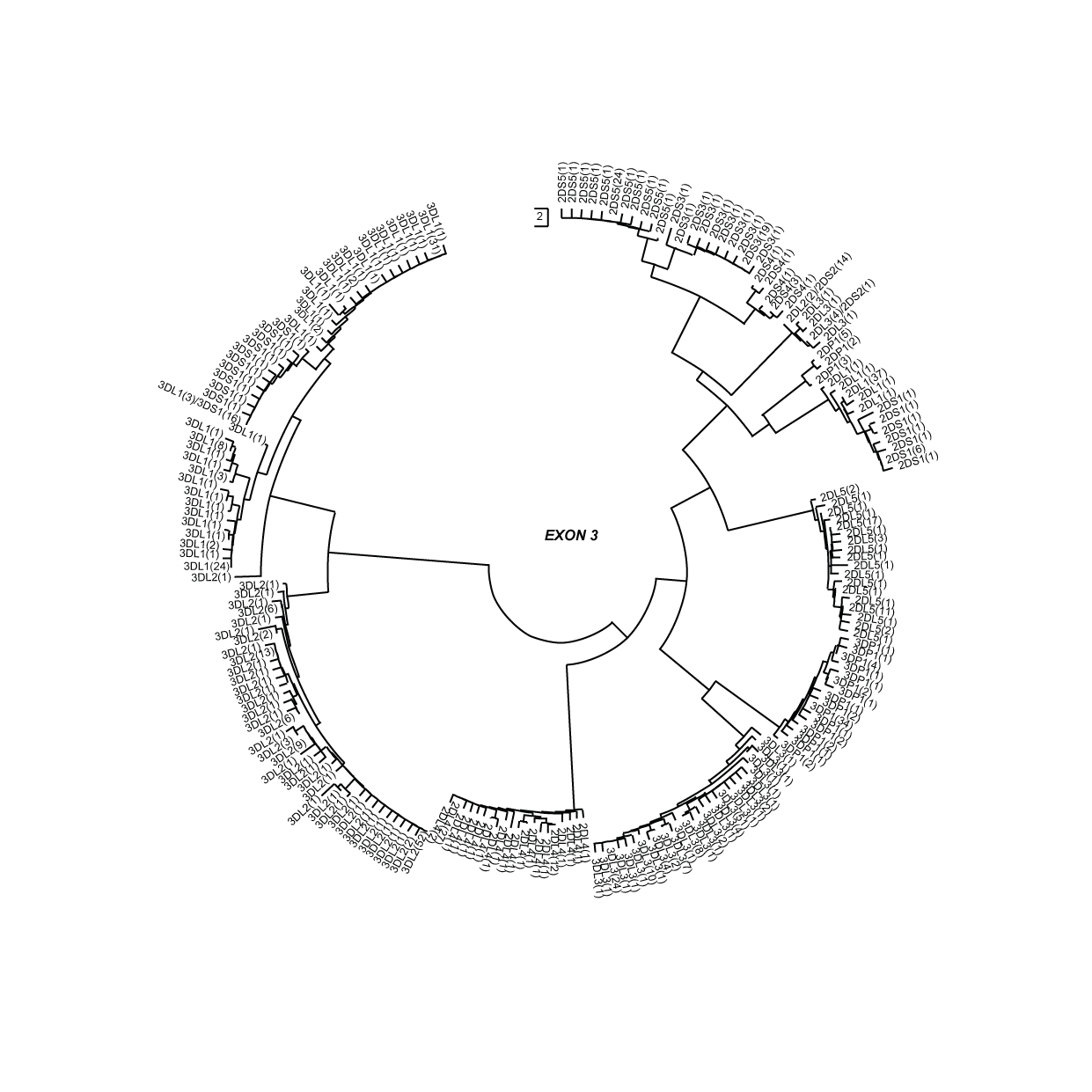


**Figure S3. Phylogenetic analysis of KIR allele sequence identity at exon 3.**


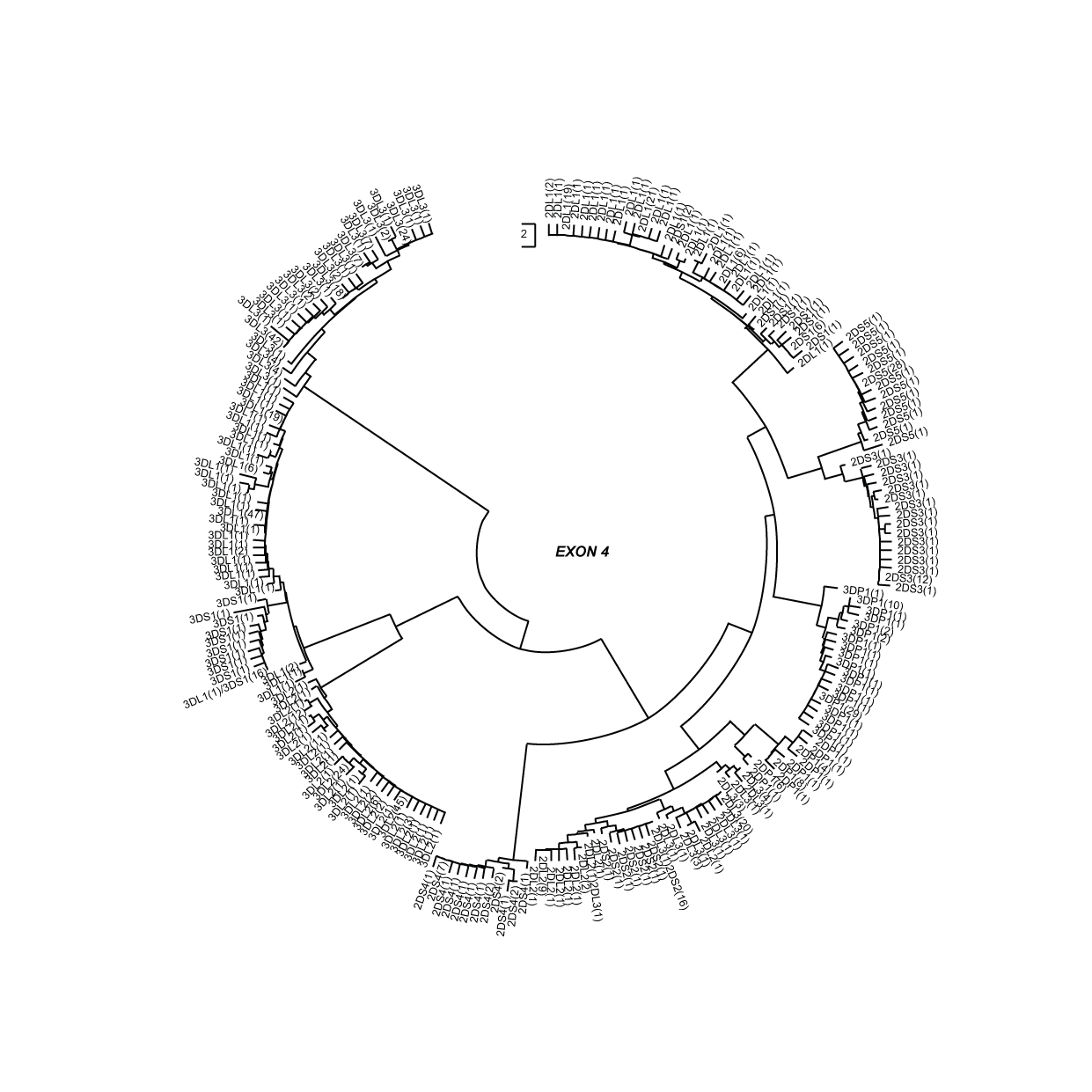


**Figure S4. Phylogenetic analysis of KIR allele sequence identity at exon 4.**


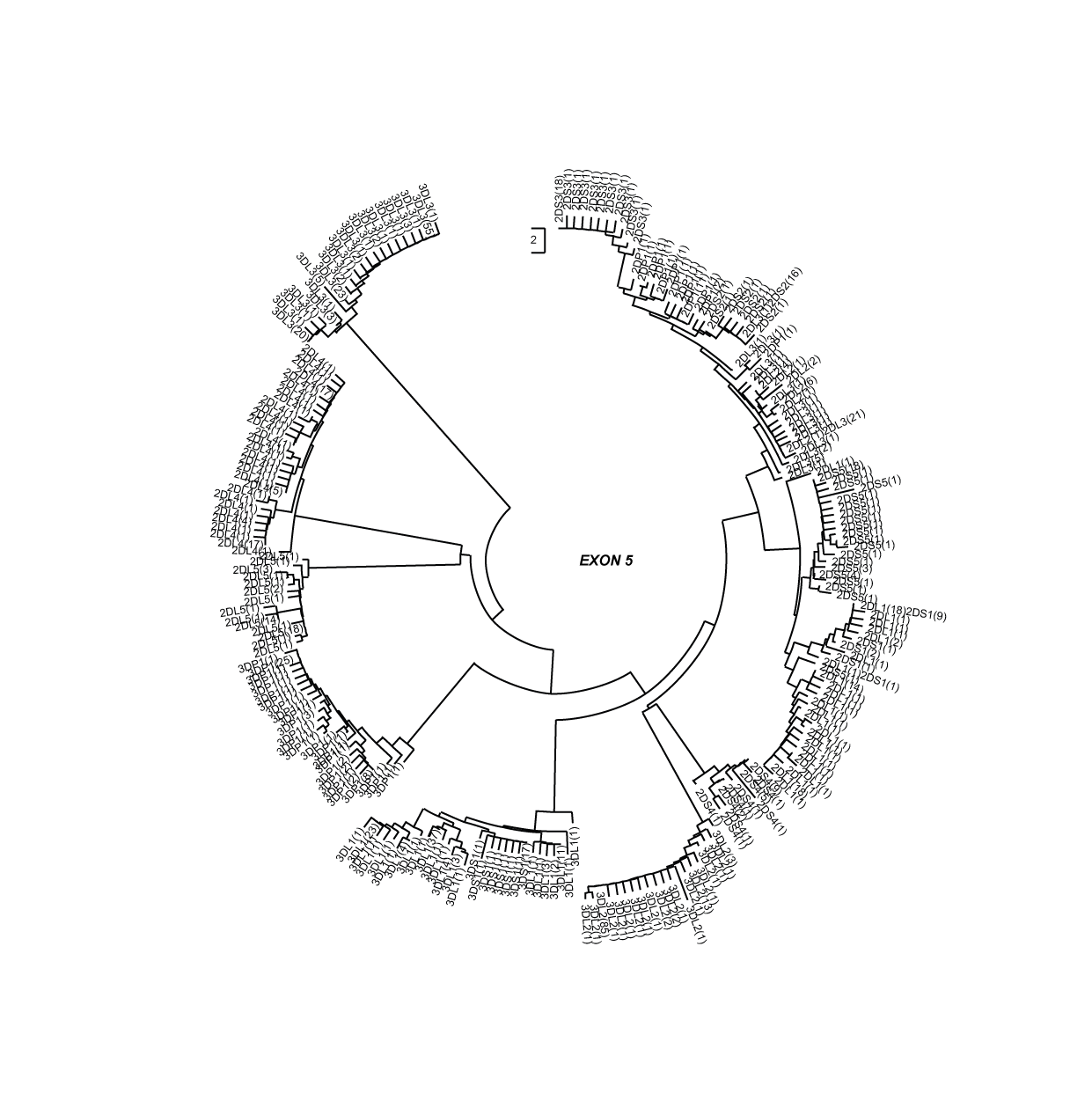


**Figure S5. Phylogenetic analysis of KIR allele sequence identity at exon 5.**


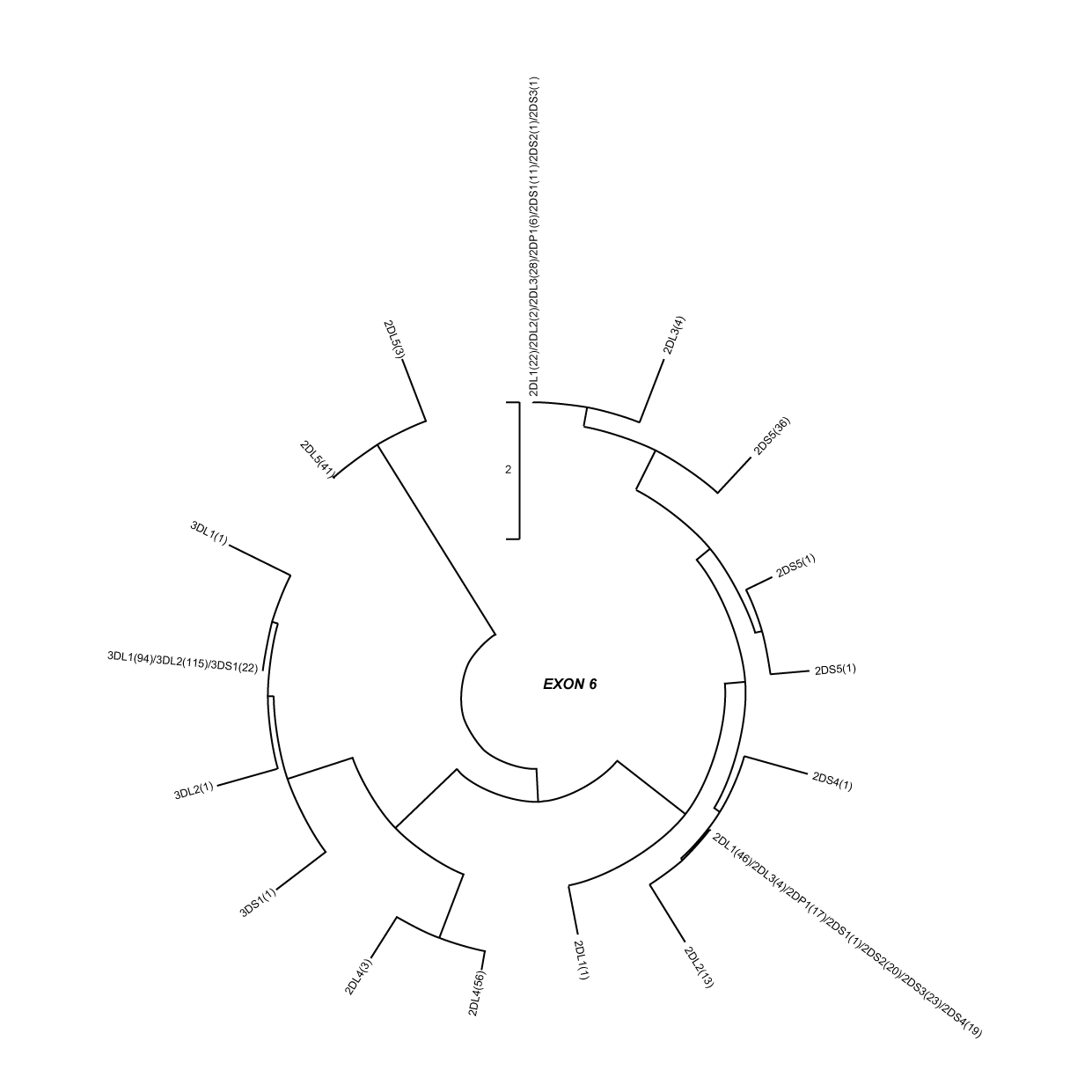


**Figure S6. Phylogenetic analysis of KIR allele sequence identity at exon 6.**


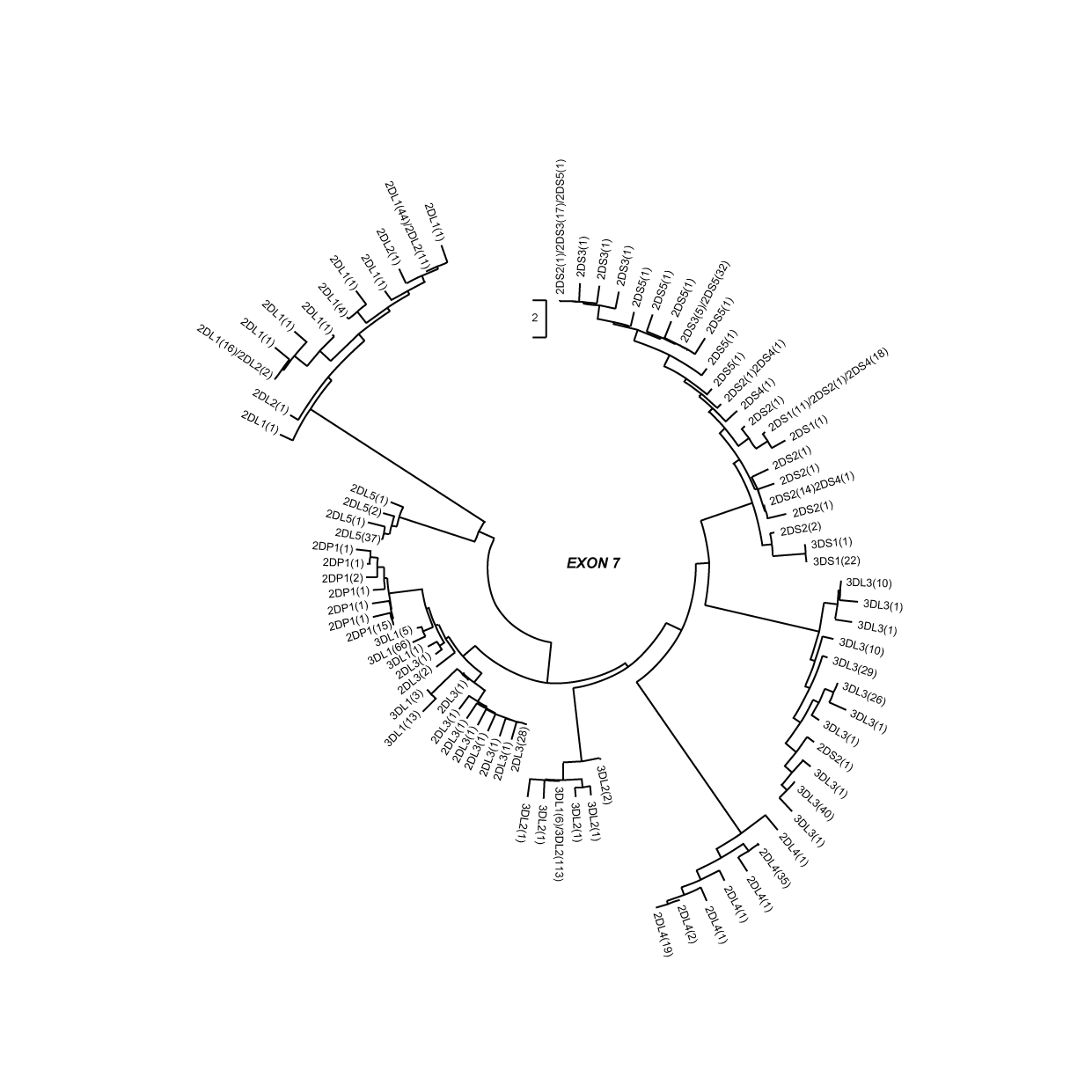


**Figure S7. Phylogenetic analysis of KIR allele sequence identity at exon 7.**


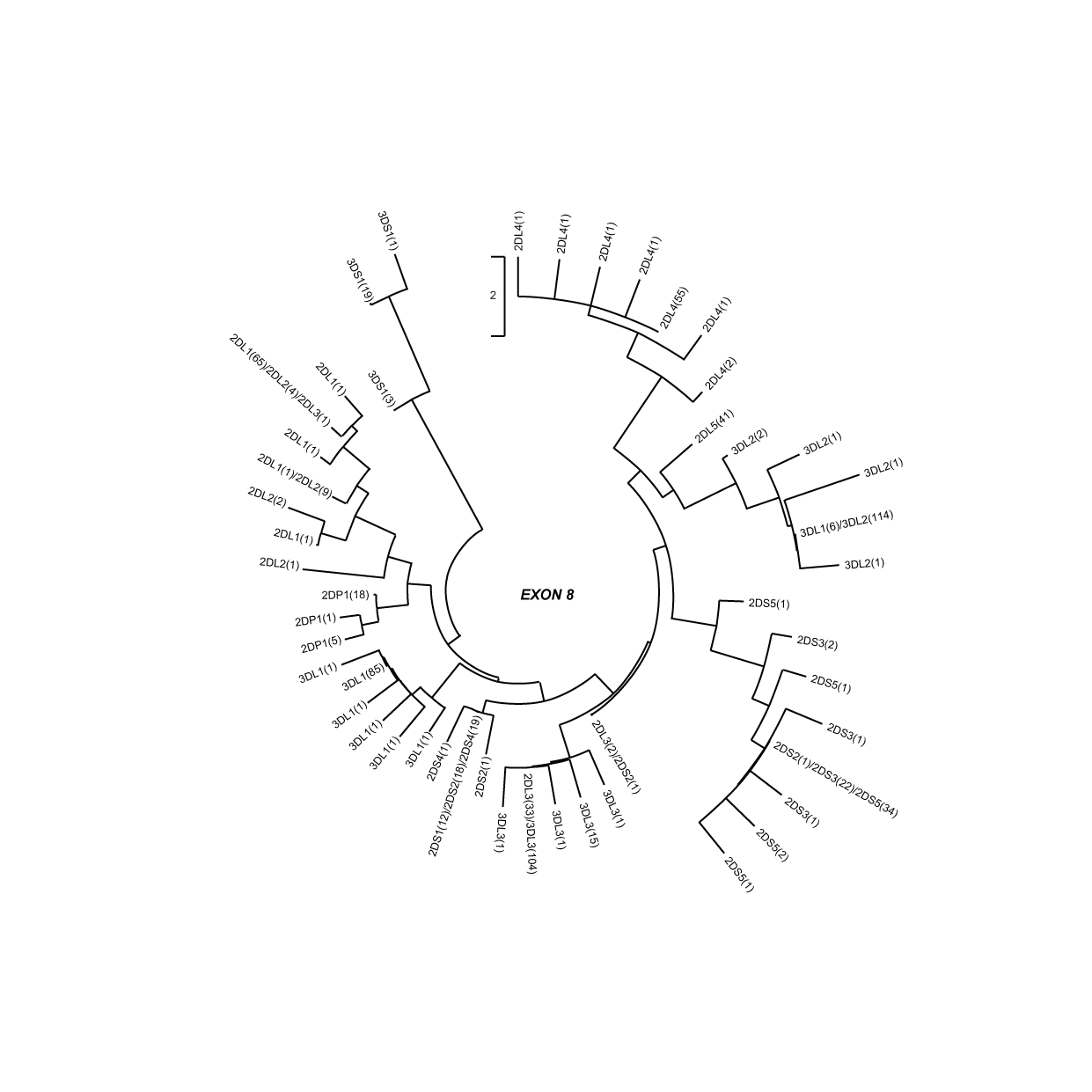


**Figure S8. Phylogenetic analysis of KIR allele sequence identity at exon 8.**


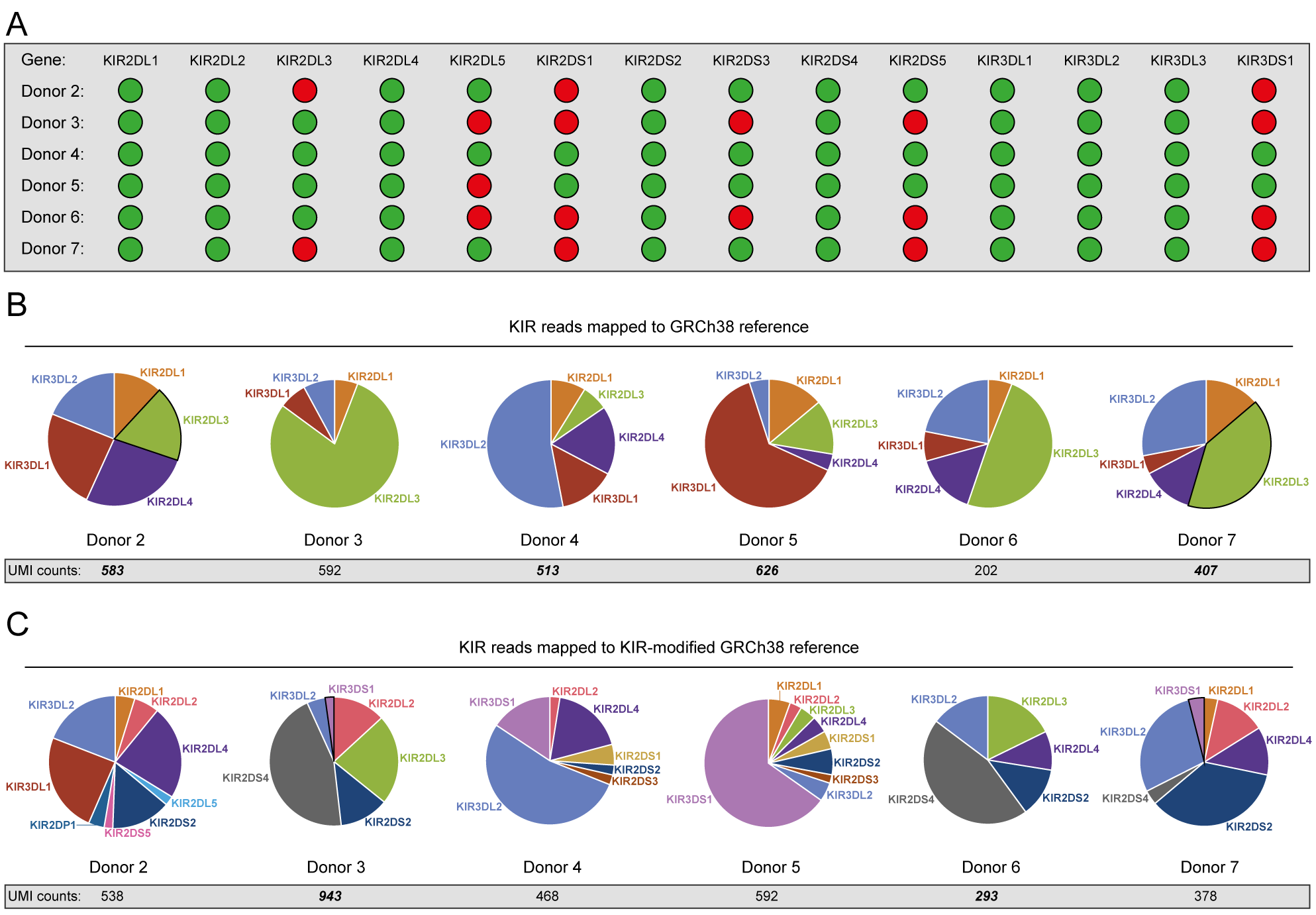


**Figure S9. Confirmation of KIR misassignment in scRNA-seq data. A)** KIR genotyping of six additional donors. Green, KIR gene is present; red, KIR gene is absent. **B)** Pie charts depicting the proportion of reads mapping to KIR loci based on the default pipeline. **C)** Pie charts representing the proportion of reads mapping to KIR loci following re-alignment to the KIR-modified GRCh38 reference. Total KIR-specific unique molecular identifier (UMI) counts are presented below each pie chart. Sections with black outline show reads incorrectly mapped to genes not in the donor’s genotype.
